# Personalized risk assessment for dynamic transition of gastric neoplasms

**DOI:** 10.1186/s12929-018-0485-6

**Published:** 2018-11-19

**Authors:** Jean Ching-Yuan Fann, Tsung-Hsien Chiang, Amy Ming-Fang Yen, Yi-Chia Lee, Ming-Shiang Wu, Hsiu-Hsi Chen

**Affiliations:** 1 Department of Health Industry Management, School of Healthcare Management, Kainan University, Taoyuan City, Taiwan; 2 Department of Internal Medicine, College of Medicine, National Taiwan University Hospital, No. 7, Chung-Shan South Road, Taipei, 10002 Taiwan; 3 Department of Integrated Diagnostics and Therapeutics, National Taiwan University Hospital, Taipei, Taiwan; 4 Graduate Institute of Clinical Medicine, College of Medicine, National Taiwan University, Taipei, Taiwan; 5 School of Oral Hygiene, College of Oral Medicine, Taipei Medical University, No. 250, Wu-Hsing Street, Xinyi District, Taipei, 110 Taiwan; 6 Institute of Epidemiology and Preventive Medicine, College of Public Health, National Taiwan University, Taipei, Taiwan; 7 Innovation and Policy Center for Population Health and Sustainable Environment, College of Public Health, National Taiwan University, Taipei, Taiwan

**Keywords:** Gastric cancer, Prevention, *Helicobacter pylori*, Endoscopy

## Abstract

**Background:**

To develop an individually-tailored dynamic risk assessment model following a multistep, multifactorial process of the Correa’s gastric cancer model.

**Methods:**

First, we estimated the state-to-state transition rates following Correa’s five-step carcinogenic model and assessed the effect of risk factors, including *Helicobacter pylori* infection, history of upper gastrointestinal disease, lifestyle, and dietary habits, on the step-by-step transition rates using data from a high-risk population in Matsu Islands, Taiwan. Second, we incorporated information on the gastric cancer carcinogenesis affected by genomic risk factors (including inherited susceptibility and irreversible genomic changes) based on literature to generate a genetic and epigenetic risk assessment model by using a simulated cohort identical to the Matsu population. The combination of conventional and genomic risk factors enables us to develop the personalized transition risk scores and composite scores.

**Results:**

The state-by-state transition rates per year were 0.0053, 0.7523, 0.1750, and 0.0121 per year from normal mucosa to chronic active gastritis, chronic active gastritis to atrophic gastritis, atrophic gastritis to intestinal metaplasia, and intestinal metaplasia to gastric cancer, respectively. Compared with the median risk group, the most risky decile had a 5.22-fold risk of developing gastric cancer, and the least risky decile around one-twelfth of the risk. The median 10-year risk for gastric cancer incidence was 0.77%. The median lifetime risk for gastric cancer incidence was 5.43%. By decile, the 10-year risk ranged from 0.06 to 4.04% and the lifetime risk ranged from 0.42 to 21.04%.

**Conclusions:**

We demonstrate how to develop a personalized dynamic risk assessment model with the underpinning of Correa’s cascade to stratify the population according to their risk for progression to gastric cancer. Such a risk assessment model not only facilitates the development of an individually-tailored preventive strategy with treatment for *H. pylori* infection and endoscopic screening but also provides short-term and long-term indicators to evaluate the program effectiveness.

## Background

Gastric cancer poses a great threat to global health that takes more than 720,000 tolls per year worldwide [1]. The current approach to gastric cancer management largely relies on endoscopic detection followed by mucosectomy, gastrectomy and/or chemotherapy; however, in the absence of early detection, gastric cancer is associated with a high fatality rate, and the 5-year survival rate for patients with locally advanced disease is only about 40% despite aggressive treatment [2].

Early detection and treatment of gastric cancer and its precancerous lesion is very feasible as carcinogenesis of gastric cancer often follows a multistage process (i.e., the Correa’s model) that develops from chronic active gastritis (CAG) to atrophic gastritis (AG), intestinal metaplasia (IM), dysplasia, and finally to carcinoma [3]. *Helicobacter pylori* is now recognized as the main risk factor that initiates this process. An estimated 89% of infection-related cancers can be prevented if *H. pylori* can be eradicated from the population of interest [4]; hence, *H. pylori* eradication is now considered the most effective way to ameliorate the burden of gastric cancer [5–7]. The age-adjusted incidence of gastric cancer has shown a steady decline, which is not only attributed to improvements in sanitation and hygiene but also to the eradication of *H. pylori* that has become a routine clinical practice in the treatment of peptic ulcers. Nevertheless, the annual number of new cases of gastric cancer in the globe is expected to still remain stable until 2030 [1]. This projection suggests universal approach to the prevention of gastric cancer may not be sufficient as the risk of developing gastric cancer varies from individual to individual and also does the acceptance of screening, compliance with the referral, and clinical workup for confirmatory diagnosis.

In the setting of mass screening, irreversible damage may already have occurred after patients have harbored *H. pylori* infection for decades before they undergo screening and treatment for *H. pylori*. This observation is supported by a recent meta-analysis, based on 8 randomized controlled trials and 16 cohort studies, of the benefit of eradication therapy; on average, gastric cancer risk was reduced only about 50% in adult patients [8]. Therefore, to efficiently eliminate the threat of gastric cancer, a population-based program should focus on both early treatment and early detection. The advent of genomics and the urgent need to prevent gastric cancer in areas with high prevalence of *H. pylori* infection and high incidence of gastric cancer have increasingly gained attention to the potential benefits of developing individually-tailored preventive strategies [9–11]. However, there is lacking of the personalized risk assessment, namely, quantitative risk-score-based stratification of the underlying population, for the development of an effective strategy that consists of *H. pylori* eradication and endoscopic screening for each individual.

Since gastric cancer is a multistep and multifactorial progressive disease, findings from basic researches should help inform the development of preventive measures [12]. Various factors may influence the transitions between stages in the development of gastric cancer, including *H. pylori* infection, genetic polymorphisms and epigenetic changes, consumption of tobacco and alcohol, and dietary habits [12, 13]. In the current study, we aimed to develop a multistep and multifactorial dynamic risk assessment model by taking into account the current evidence on environmental, genetic, and epigenetic risk factors responsible for gastric carcinogenesis. We also provided short-term (such as premalignant gastric lesions) and long-term (such as incidence and mortality of gastric cancer) indicators to support the effectiveness when such a personalized prevention program was implemented on a high-risk population.

## Methods

### Evolution of community-based prevention campaign on Matsu Islands

There are three phases of community-based prevention program gradually offered for residents on the Matsu Islands, an island archipelago located in the Taiwan Strait (also an offshore island between Taiwan and China). The residents had a high gastric cancer burden, with an incidence rate 3–5 folds higher than that of the main island of Taiwan and the highest mortality rate from gastric cancer among all Taiwanese populations. Therefore, a two-stage screening program targeting the premalignant gastric lesions and early-stage gastric cancer was conducted in 1996–1998 using the serum anti-*H. pylori* immunoglobin G antibody test and serum pepsinogen measurement as the first stage and those who tested positive were referred to the second-stage endoscopy for confirmatory diagnosis and histological assessment; the results have been described in full elsewhere [10]. The second phase was to launch a community-based integrated screening since 2002 onwards with five common cancers in combination with other examinations for chronic diseases [14]. The program invited residents aged 30 and above on the Matsu Islands to participate annually with various inter-screening intervals for different items. The third phase was to introduce a chemopreventive program for gastric cancer by using the mass eradication of *H. pylori* infection since 2004 [9, 11]. The effects of *H. pylori* infection and conventional risk factors were estimated from the empirical data collected from three phases of the community-based screening programs.

### Gastric cancer prevention programs

As the current paper places emphasis on the prevention of gastric cancer, here we detail the evolution of prevention programs for gastric cancer. In 1996–1998, a screening program mainly based on the serological biomarkers was conducted. The first stage included the serum anti-*H. pylori* immunoglobin G antibody test and serum pepsinogen measurement. Those with positive results in the first stage were referred to confirmatory endoscopy and histological assessment. Among 3541 residents aged 30 years or older registered in population list, a total of 2184 residents participated in the first stage of the screening project. Among 946 who had first-stage positive results, 523 complied with second-stage endoscopic examination, 325 underwent endoscopic biopsy for histological evaluation, and 2 gastric cancers were detected endoscopically.

The second gastric cancer prevention program was launched in 2004, which included the first stage with C^13^-urea breath test and the second stage with endoscopic examination and histological evaluation. In 2004, a total of 4121 participants participated and 2598 (63%) tested positive for *H. pylori* infection. Endoscopy was done for 1762 *H. pylori* carriers for histological assessment and 4 gastric cancers were found. Histology was classified using the updated Sydney system [15]. The overall eradication rate was 97.7% following 2 courses of antibiotic treatments.

**Fig. 1:**
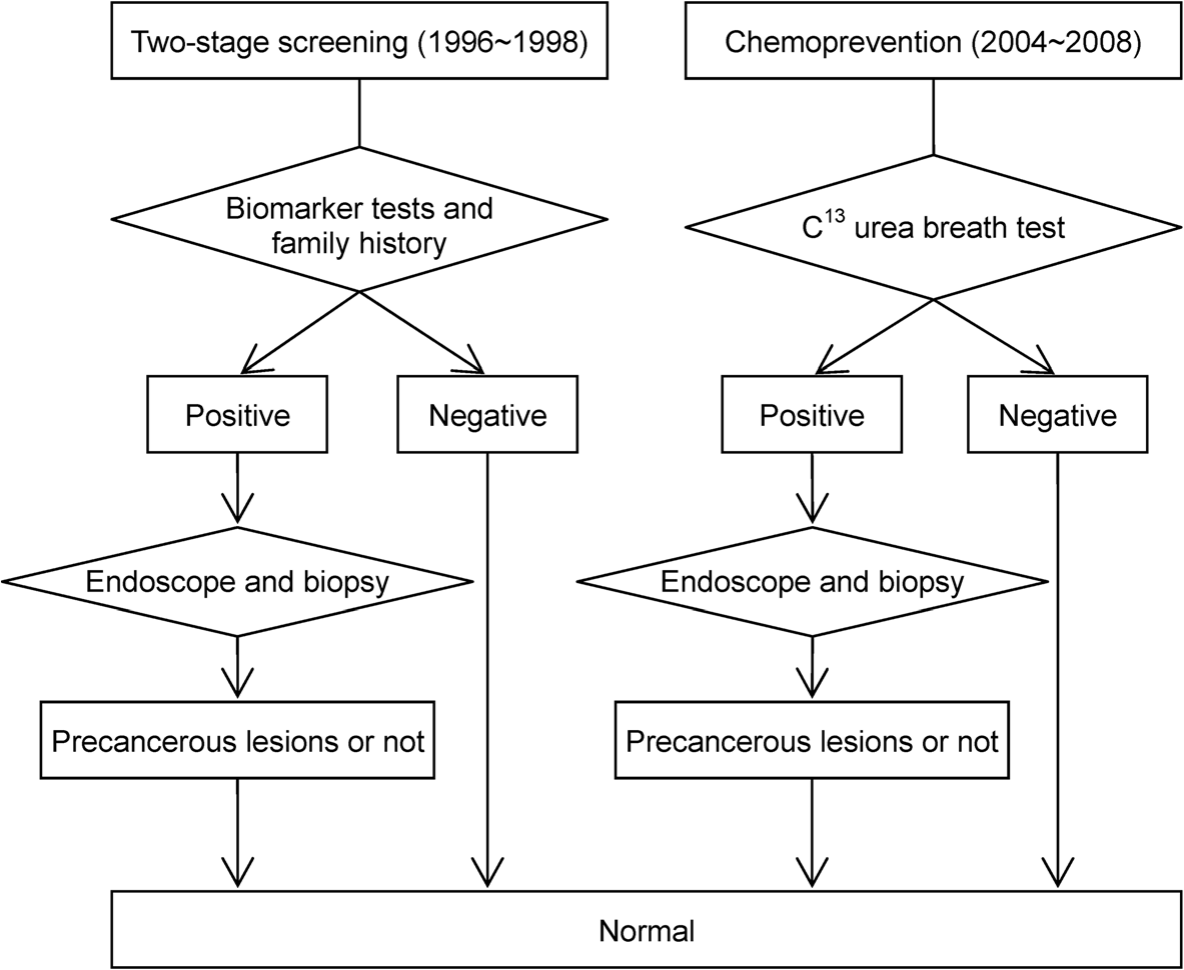
The flowchart for the gastric cancer screening programs in the Matsu Islands

The study flow chart for collecting information on this cohort is depicted in Fig. 1. Because these two programs were in conjunction with a community-based integrated screening program, in addition to the transition between states (normal → CAG, CAG → AG, AG → IM, and IM → gastric cancer), information on the state-specific risk factors, such as the demographic data, lifestyle factors, diet habits, and family and medical histories, were available. Further searching for the information of genetic susceptibility and genetic/epigenetic alternations from literature, we can build up the following personalized multistate risk assessment model.

### Personalized multistate risk assessment model

We constructed a multistep and multifactorial disease natural history in the light of the Correa’s model that can be delineated as follows: normal → CAG → AG → IM → gastric cancer [3], superimposed with state-specific factors in each state transition. The relative risk of *H. pylori* infection, history of upper gastrointestinal disease, exercise habit, fruit intake, chicken intake, dry fish intake, and salt fish intake on different transitions were estimated on the basis of the empirical data from the Matsu Islands [10, 11, 13, 14]. The relative risks associated with genetic and epigenetic factors were extracted from the literature and were fitted with empirical data [16–21].

**Fig. 2:**
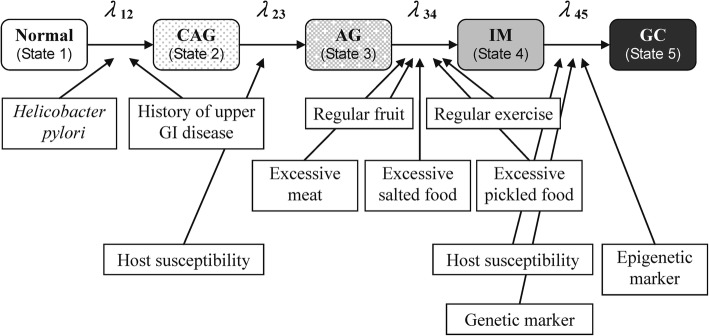
The multistep and multifactorial progression of gastric cancer. For example, regular consumption of fruit and regular exercise may alleviate the transition from atrophic gastritis to intestinal metaplasia. Abbreviations: CAG: chronic active gastritis; AG: atrophic gastritis; IM: intestinal metaplasia; GC: gastric cancer

Figure 2 shows the five-state Markov model for gastric cancer. In the light of recognized risk factors, we calculated the incidences of the transition from normal to CAG (λ_12_), the transition from CAG to AG (λ_23_), from AG to IM (λ_34_), and from IM to gastric cancer (λ_45_), associated with the corresponding relevant risk factors in the proportional hazard form as shown in the following equations:

$$ {\uplambda}_{12}={\uplambda}_{120}\times \mathit{\exp}\left({\beta}_1\times (HP)+{\beta}_2\times \left( Upper\  GI\  disease\right)\right) $$

$$ {\uplambda}_{23}={\uplambda}_{230}\times \mathit{\exp}\left({\beta}_3\times \left( IL1 RN\ 2/2\right)\right) $$

$$ {\uplambda}_{34}={\uplambda}_{340}\times \mathit{\exp}\left({\beta}_4\times (Exercise)+{\beta}_5\times \left( Fruit\ input\right)+{\beta}_6\times \left( Meat\ input\right)+{\beta}_7\times \left( Picked\ food\ input\right)+{\beta}_8\times \left( Salty\ food\ input\right)\right) $$

$$ {\uplambda}_{45}={\uplambda}_{450}\times \mathit{\exp}\left({\beta}_9\times (p53)+{\beta}_{10}\times \left(\mathrm{E}-\mathrm{cadherin}\ 160\  AA, CA\right)+{\beta}_{11}\times \left( MTHFR\ 677\  TT\right)+{\beta}_{12}\times (MSI)+{\beta}_{13}\times (LOX)+{\beta}_{14}\times \left(p41 ARC\right)\right) $$

These four regression models are used for the development a personalized risk assessment model for deriving four transition risk scores for normal → CAG, CAG → AG, AG → IM, and IM → cancer, and also the composite score by combining four transition risk scores with the assignment of different weights to each transition risk score. The weights assigned to each transition (normal → CAG, CAG → AG, AG → IM, and IM → cancer) were based on the relative value of taking the logarithm of baseline rate for the three transitions (normal → CAG, CAG → AG, and IM → cancer) in comparison with the reference group (AG → IM).

### Computer simulation of the individual risk

We simulated a cohort of 100,000 subjects aged 30–79 years who were followed up for 10 years in order to generate the 10-year cumulative risk of developing gastric cancer. The infection with *H. pylori*, history of upper gastrointestinal disease, lifestyle, and dietary habits of this hypothetic cohort were determined by assigning the distributions of the cohort on the Matsu Islands. The rates of P53 codon 72 polymorphisms, E-cadherin-160A polymorphisms, microsatellite instability (MSI), and the methylation levels of LOX and p41ARC were also derived. The cohort was therefore classified into different risk groups according to deciles of the composite risk score as mentioned above.

### Statistical analysis

A continuous-time five-state Markov process in the light of the Correa’s model was developed by defining four transition rates as mentioned above into the intensity 5 × 5 matrix form. The transition probabilities during time *t* in terms of matrix for each transition were also derived by using the forward Kolmogorov equations. Given the Markov property that the disease status in each year for any individual was dependent on his/her disease status in the previous year but independent of the disease status previously, the log likelihood function was developed by using the available empirical data on each transition mode, respectively, including normal → CAG, CAG → AG, AG → IM, and IM → cancer, to estimate four transition parameters and also the state-specific regression coefficients. The latter was formed as the basis for the development of transition risk score and composite risk score. All analyses were done using SAS software (version 9.4; SAS Institute, Cary, NC, USA).

## Results

### State-specific correlates associated with the Correa’s multistate model

The state-by-state transition rates per year were 0.0053, 0.7523, 0.1750, and 0.0121 per year from normal to CAG, CAG to AG, AG to IM, and IM to gastric cancer, respectively. Table 1 shows the both effects of *H. pylori* infection and histories of upper gastrointestinal disease on the incidence of CAG; the effect of genetic susceptibility on the transition from CAG to AG; the effects of lifestyle and dietary habits on the transition from AG to IM; and the effects of genetic susceptibility, microsatellite instability, and DNA methylation level (epigenetic factor) on the transition from IM to gastric cancer. Accordingly, the transition rates between states before the development of invasive gastric cancer can be expressed as:

$$ {\uplambda}_{12}=0.001232\times \mathit{\exp}\left(1.7733\times (HP)+1.0682\times \left( Upper\  GI\  disease\right)\right) $$

$$ {\uplambda}_{23}=0.6838\times \mathit{\exp}\left(0.8198\times \left( IL1 RN\ 2/2\right)\right) $$

$$ {\uplambda}_{34}=0.1536\times \mathit{\exp}\left(-0.4463\times (Exercise)-0.5276\times \left( Fruit\ input\right)+0.7178\times \left( Meat\ input\right)+0.8629\times \left( Intake\ of\ prickled\ food\right)+1.1184\times \left( Intake\ of\ salty\ food\right)\right) $$

$$ {\uplambda}_{45}=0.0005\times \mathit{\exp}\left(-0.1744\times \left(p53\  Arg, Arg\right)-0.2107\times \left(\mathrm{E}-\mathrm{cadherin}\ 160\  AA, CA\right)+0.4947\times \left( MTHFR\ 677\  TT\right)+1.1282\times (MSI)+0.8629\times (LOX)+1.3137\times \left(p41 ARC\right)\right) $$

**Table 1:** The multifactorial effects of *H. pylori* infection, upper gastrointestinal disease, life style and dietary habit, genetic and epigenetic factors on the multistep progression of gastric cancer

Variables	Estimate	95% CI	References
Effect on transition from normal to CAG			
Transition rate (λ_120_)	0.0053	0.0051–0.0056	[10, 11]
RR of *H. pylori infection*	5.89	5.63–6.17	[10, 11]
RR of history of upper gastrointestinal disease	2.91	2.76–3.06	[10, 11]
Effect on transition from CAG to AG			
Transition rate (λ_230_)	0.7523	0.7071–0.7975	[10, 11]
RR of interleukin-1 RN VNTR polymorphism: 2/2 genotype	2.27	1.40–3.70	[14]
Effect on transition from AG to IM			
Transition rate (λ_340_)	0.1750	0.1640–0.1860	[10, 11]
RR of regular exercise	0.64	0.59–0.68	[10, 11]
RR of frequent fruit	0.59	0.53–0.65	[10, 11]
RR of frequent meat	2.05	1.82–2.30	[10, 11]
RR of frequent prickled food	2.37	1.86–3.02	[10, 11]
RR of frequent salty food	3.06	2.41–3.89	[10, 11]
Effect on transition from IM to GC			
Transition rate (λ_450_)	0.0121	0.0097–0.0145	[10, 11]
RR of p53 codon 72 polymorphism: Arg/Arg genotype	0.84	0.72–0.99	[15]
RR of E-cadherin (*CHD1*)-160A polymorphism: AA/CA genotype	0.81	0.67–0.99	[16]
RR of MTHFR 677 polymorphism: TT genotype	1.64	1.36–1.97	[17]
RR of microsatellite instability	3.09	2.79–3.42	[18]
RR of methylation level of *LOX*	2.37	2.17–2.59	[19]
RR of methylation level of *p41ARC*	3.72	3.29–4.21	[19]

Abbreviations: *RR* relative risk, *CI* confidence interval, *CAG* chronic active gastritis *AG* atrophic gastritis, *IM* intestinal metaplasia, *GC* gastric cancer, *RR* relative risk, *VNTR* variable number tandem repeat, *MTHFR* methylenetetrahydrofolate reductase

According to these four transition rates, four corresponding transition risk scores for serial state transitions are developed by using their regression coefficients:

$$ \mathrm{Score}\ \left(\mathrm{normal}\to \mathrm{CAG}\right)=\left\{1.7733\times (HP)+1.0682\times \left( Upper\  GI\  disease\operatorname{}\right)\right\} $$

$$ \mathrm{Score}\ \left(\mathrm{CAG}\to \mathrm{AG}\right)=\left\{0.8198\times \left( IL1 RN\ 2/2\right)\right\} $$

$$ \mathrm{Score}\ \left(\mathrm{AG}\to \mathrm{IM}\right)=\left\{-0.4463\times (Exercise)-0.5276\times \left( Fruit\ input\right)+0.7178\times \left( Meat\ input\right)+0.8629\times \left( Prikled\ food\ input\right)+1.1184\times \left( Salty\ food\ input\right)\right\} $$

$$ \mathrm{Score}\ \left(\mathrm{IM}\to \mathrm{cancer}\right)=\left\{\left(-0.1744\times \left(p53\  Arg, Arg\right)-0.2107\times \left(\mathrm{E}-\mathrm{cadherin}\ 160\  AA, CA\right)+0.4947\times \left( MTHFR\ 677\  TT\right)+1.1282\times (MSI)+0.8629\times (LOX)+1.3137\times \left(p41 ARC\right)\right)\right\} $$

The composite score in the light of four transition risk scores was also developed by assigning different weights to each transition risk score. The weights assigned to normal → CAG, CAG → AG, AG → IM, and IM → cancer were 15, 1, 5, and 20 based on the relative value of taking the logarithm of baseline rate for three baseline transitions in comparison with the reference group (AG → IM).

### Kinetic epidemiological curves of the multistate outcomes

**Fig. 3:**
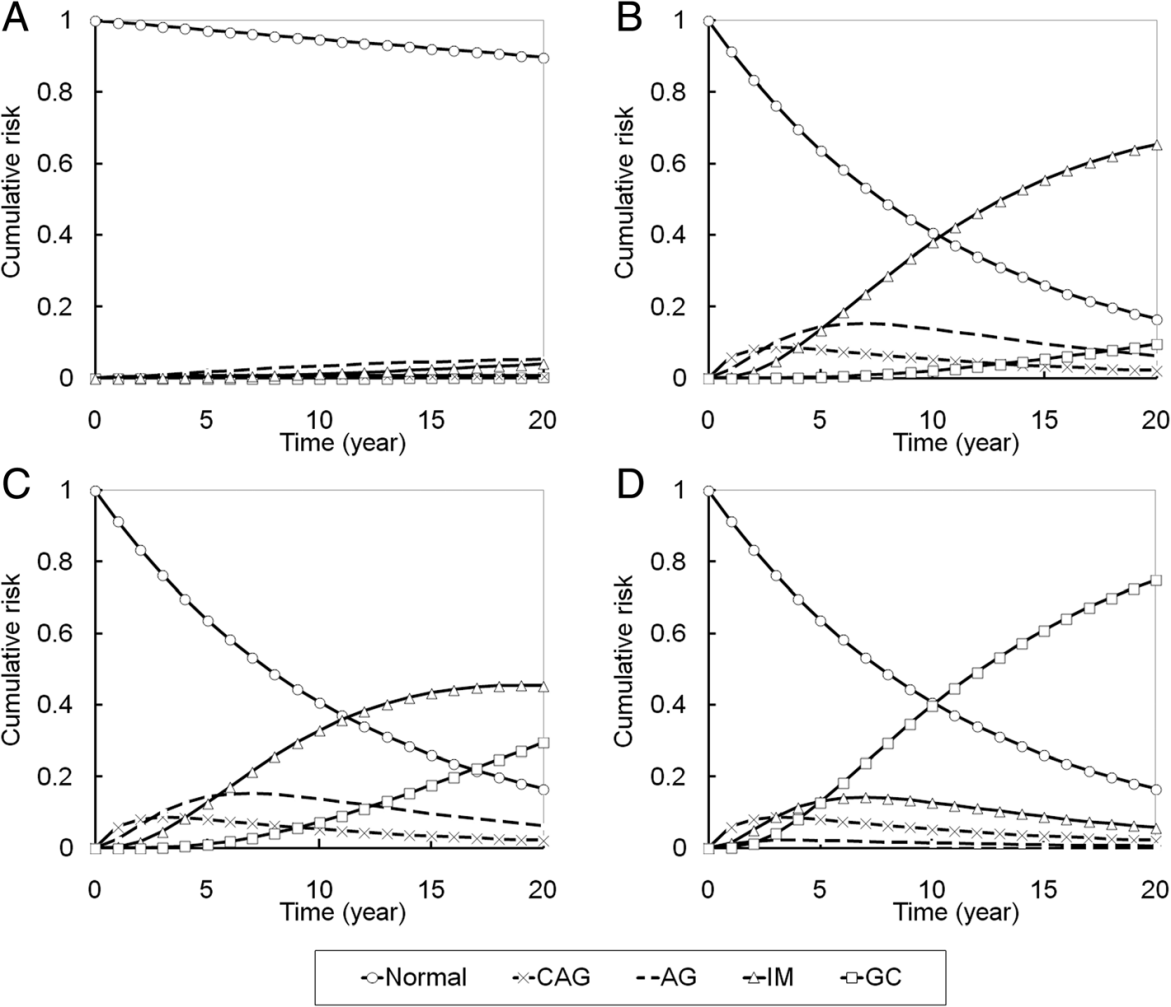
The 20-year cumulative risk of multi-state outcomes of gastric neoplasms for four hypothetical subjects with: (**a**) low risk (without *H. pylori* infection, with regular exercise and fruit intake, p53 codon 72 Arg/Arg, E-cadherin C/C, MSI stable, and methylation levels LOX/p41 ARC: 7.2%/6.2%); (**b**) intermediate risk (with *H. pylori* infection/upper gastrointestinal disease history/large intake of meat, without regular exercise and fruit intake, p53 codon 72 Pro/Pro, E-cadherin A/A, MSI stable, and methylation LOX/p41 ARC: 7.2%/6.2%); (**c**) high risk (with *H. pylori* infection, upper gastrointestinal disease history, smoking, large intake of meat, without regular exercise or fruit intake, p53 codon 72 Pro/Pro, E-cadherin A/A, MSI stable, and methylation LOX/p41 ARC: 7.2%/11.2%); and (**d**) extremely high risk (with *H. pylori* infection, upper gastrointestinal disease history, smoking, large intake of meat, intake of salty fish and dry fish, without regular exercise or fruit intake, p53 codon 72 Pro/Pro, E-cadherin A/A, MSI instable, and with methylation levels: LOX/p41 ARC: 12.2%/11.2%). Abbreviations: CAG: chronic active gastritis; AG: atrophic gastritis; IM: intestinal metaplasia; GC: gastric cancer

The kinetic epidemiological curves of multi-state outcomes (from normal to gastric cancer) for four hypothetical subjects at the low risk, intermediate risk, high risk, and extremely high risk are depicted in Fig. 3. For example, low risk could be defined as no *H. pylori* infection, with regular exercise and fruit intake, p53 codon 72 Arg/Arg, E-cadherin C/C, MSI stable, and methylation levels LOX/p41 ARC: 7.2%/6.2%. Intermediate risk was defined as having *H. pylori* infection, a history of upper gastrointestinal disease, large intake of meat, no regular exercise or fruit intake, p53 codon 72 Pro/Pro, E-cadherin A/A, MSI stable, and methylation LOX/p41 ARC: 7.2%/6.2%. High risk was defined as having *H. pylori* infection, a history of upper gastrointestinal disease, smoking, large intake of meat, no regular exercise or fruit intake, p53 codon 72 Pro/Pro, E-cadherin A/A, MSI stable, and methylation LOX/p41 ARC: 7.2%/11.2%. Extremely high risk was defined as having *H. pylori* infection, a history of upper gastrointestinal disease, smoking, large intake of meat, intake of salty fish and dry fish, no regular exercise or fruit intake, p53 codon 72 Pro/Pro, E-cadherin A/A, MSI instable, and methylation levels: LOX/p41 ARC: 12.2%/11.2%.

For the intermediate risk condition relative to the low risk, the cumulative risk for intestinal metaplasia increased significantly through time. For the high risk condition, although the cumulative risk for intestinal metaplasia increased less than for the intermediate risk condition, gastric cancer was more likely to develop. For the extremely high risk condition, the cumulative risk for gastric cancer development was up to 40% after 10 years.

Not only the different risk profile of an individual, Fig. 3 also demonstrates the pattern of dynamic transition from normal to the occurrence of gastric cancer**.** These curves provide a basis for the development of surrogate endpoint and primary endpoint for the evaluation of a personalized prevention program.

### Personalized risk assessment for gastric cancer

We classified our cohort into four risk groups (low, intermediate, high, and extremely high) and deciles of risk and calculated the 10-year and lifetime risk of developing gastric cancer by using composite risk score (Table 2). Compared with the median risk group, the most risky decile had a 5.22-fold risk of developing gastric cancer, and the least risky 5% around one-twelfth of the risk. The median 10-year risk for gastric cancer incidence and mortality were 0.77 and 0.53%, respectively. The median lifetime risk for gastric cancer incidence and mortality were 5.43 and 5.06%, respectively. By decile, the 10-year risk ranged from 0.06 to 4.04% and the lifetime risk ranged from 0.42 to 21.04%.

**Table 2:** The 10-year and lifetime risk of developing gastric cancer by using the composite risk score

Risk group	RR	10-year risk	Life-time risk
95–100	5.22	4.04%	21.04%
80–95	3.80	2.94%	16.50%
60–80	2.70	2.09%	13.01%
51–60	1.23	0.95%	7.30%
Median (50)	1.00	0.77%	5.43%
40–49	0.92	0.71%	4.64%
30–40	0.17	0.13%	0.98%
5–30	0.08	0.06%	0.53%
0–5	0.08	0.06%	0.42%

Abbreviations: *RR* relative risk

## Discussion

### Personalized dynamic risk assessment model for gastric cancer

Environmental risk factors and biological markers (including the genetic and epigenetic determinants) related to the progression from premalignant gastric lesions to gastric cancer provide an insight into the benefits of a prophylactic intervention and screening program. The development of an individually-tailored method to stratify the risk of multistate disease outcome for the underlying population plays an important role for the planning of personalized preventive strategies for gastric cancer. However, how to develop a systematic framework for such a purpose has been barely addressed before. In this article, we demonstrate how to develop a multistep and multifactorial risk assessment model, taking into account environmental, genetic, and epigenetic factors, and to produce a risk-score-based stratification upon which we can develop individualized prevention strategies to reduce the incidence and mortality of gastric cancer.

The personalized risk assessment model with the incorporation of personal characteristics and possible biological markers here provides a new insight into how to integrate genetic counseling, epidemiology, health information, and healthcare management into a unifying framework based on the risk of developing premalignant gastric lesion and gastric cancer, and throws light on how to develop an individual-tailored approach. For the risk assessment of gastric cancer, the currently available blood tests mainly include the serum PG, which has long been considered a reliable biomarker of the functional and morphologic status of the gastric mucosa [22]. A meta-analysis of 1520 patients with gastric cancer and 27,723 controls showed a sensitivity of 70%, a specificity of 79%, and a positive likelihood ratio of 3.3 of the combination of PG-I level and the PG-I/II ratio to detect gastric cancer [23]. Also in our study population, a previous study has shown that a low serum PG-I level and/or a low PG-I/II ratio were predictive of a higher risk of gastric cancer death following 16 years of follow-up [24]. Nonetheless, the subjects with abnormal PG level was found only associated with about 3–4 folds of gastric cancer risk; therefore, the predictability of using this serological marker remains limited as the phenotypes of gastric cancer may include the intestinal and the diffuse types, which may be in turn associated with different patterns of the genetic and epigenetic alternations. Also because PG testing only detects atrophic gastritis coexisting with cancer, about one third of gastric cancer cases (the diffuse type) may be missed by using the PG teste as a non-endoscopic biomarker for the detection of gastric cancer.

### Comparison with the universal screening approaches

Several studies have indicated that the secondary prevention with endoscopic screening could decrease the mortality from gastric cancer. In the Korean Nationwide Screening Program, those who received endoscopic screening were associated with 47% reduction of death from gastric cancer [25]. A synthetic study that included 6 cohort studies and 4 case control studies in Asia (comprising 342,013 individuals) consistently showed a 40% reduction in gastric cancer mortality [26]. However, such a universal endoscopy approach greatly relied on the capacity of endoscopists and failed to decrease the incidence of gastric cancer. On the other way, the primary prevention through *H. pylori* eradication has greatly attracted attention as the strategy for gastric cancer prevention on a population-wide scale [1, 6, 27], which was supported by a consensus meeting that formally declared *H. pylori* gastritis as an infectious disease, which should be treated and cured [28]. In our previous meta-analysis that included 715 incident gastric cancers among a total of 48,064 individuals/340,255 person-years (also included the population on Matsu Islands), individuals with eradication of *H. pylori* infection had a reduction of 47% in gastric cancer risk than those who did not receive eradication therapy [8]. The magnitude of risk reduction related to *H. pylori* eradication would be greater in populations with the more aggressive *H. pylori* strains and the higher percentage of genetic trait that is more susceptible to carcinogens, which may lead to more intensive host-bacterial interaction, the more rapid carcinogenic process, and thus the higher risk of gastric cancer. Therefore, in high-risk populations, in addition to the intensive endoscopic surveillance, the combination of a mass eradication program is highly desirable.

### The personalized preventive approach

In recent years, many genetic and epigenetic markers have been reported as promising biomarkers to predict and stratify gastric cancer risk [29–31]; however, none of them has been implemented on the population level. Our study highlights a method illustrating how to apply these novel biomarkers to a high-risk population that has initiated the mass eradication program, which has a significant implication for how to integrate the primary and secondary prevention strategies to maximize the benefit from a screening program and optimize the allocation of limited endoscopic resources. Such a personalized risk assessment model is very helpful for the development of personalized preventive strategies. According our proposed models, the median risk group might start screening in middle age with medium-range intervals, in late age with longer intervals for those at lower risk, and those at extremely high risk might start screening programs at younger ages with the shortest interval. The same logic can be applied to chemoprevention on age to commence.

It is noteworthy that such a personalized risk assessment model with multistep and multifactorial property also provides an opportunity of point-of-care for the dynamic transition of gastric cancer with personal viewpoint, which also makes major contribution to shared decision-making for personalized prevention for gastric cancer.

### Short-term and long-term evaluations

Despites the advantage of using personalized strategies, evaluation of its effectiveness is intractable partly because of enormous costs and time in longitudinal follow-up study and partly because of the complex design of personalized strategy. The proposed dynamic multistate model with the underpinning of Correa’s cascade may be a panacea. The Fig. 3 shows dynamic transition from normal to gastric cancer through the premalignant gastric lesions. Such a kinetic epidemiological curve provides an opportunity for the development of short-term indicators, such as AG and IM, and long-term indictors, such as incidence and mortality of gastric cance**r.**

### Strengths and limitations of the study

Our strengths include the use of a cohort study design in a high-risk community with gastric cancer, which reduces the possibility of selection bias common in previous studies. A cohort with comprehensive demographic characteristics, baseline *H. pylori* infection status, and histological assessment presented a unique opportunity to estimate state-to-state transition rates accurately. We also simulated the clinical scenarios about how to implement such a risk-score-based stratification on the population level, which may be of great information for the healthcare policy makers to develop a policy that consists of individual risk profiles such that the incidence and mortality of gastric cancer can be efficiently reduced especially for the high-risk populations and the limited medical resources can be properly allocated.

However, there are several limitations in this study. First, gastric cancer is a heterogeneous disease. Molecular heterogeneity has been shown through the existence of subtypes that differ in histopathology and anatomic site, gene expression, DNA methylation, and oncogenic pathways [12]. Although the five-state Markov model and the resulting equations could not account for all potential genetic/epigenetic risk factors, the concept of risk-score-based stratification could provide a specific prevention strategy for high risk individuals to reduce their incidence and mortality rates of gastric cancer. Second, the development of dietary habits is highly dependent on the underlying culture and socioeconomic status of an individual. The present cohort was surveyed on dietary items in the 1990s. We found that most of the habits pertaining to the intake of salted foods were time-invariant, whereas the intake of meat, fruit, milk, and shrimp sauce were time-variant [13]. Therefore, whether our model based on the nutritional factors at the initial stage could be applied to the more modern population deserves further observation.

## Conclusions

We demonstrate how to develop a personalized Correa gastric cancer model to stratify the risk of developing premalignant gastric lesions and gastric cancer using clinical and genomic factors. The proposed personalized risk assessment model provides a new insight into health planning for the development of preventive strategies regarding the eradication of *H. pylori* infection and early detection with endoscopy with short-term endpoints to reduce the premalignant gastric lesions and with long-term endpoints to reduce the incidence and mortality of gastric cancer.

## Abbreviations

AG: Atrophic gastritis
CAG: Chronic active gastritis
IM: Intestinal metaplasia
MSI: Microsatellite instability
MTHFR: Methylenetetrahydrofolate reductase
PG: Pepsinogen
RR: relative risk
VNTR: Variable number tandem repeat
